# Assessment of early diabetic retinopathy severity using ultra-widefield Clarus versus conventional five-field and ultra-widefield Optos fundus imaging

**DOI:** 10.1038/s41598-023-43947-5

**Published:** 2023-10-10

**Authors:** Yuanyuan Xiao, Handong Dan, Xiaofeng Du, Michel Michaelide, Xiaodong Nie, Wanxiao Wang, Miao Zheng, Dongdong Wang, Zixu Huang, Zongming Song

**Affiliations:** 1 Henan Provincial People’s Hospital, Henan Eye Institute, Henan Eye Hospital, Henan Key Laboratory of Ophthalmology and Visual Science, People’s Hospital of Zhengzhou University, People’s Hospital of Henan University, No. 7 Weiwu Road, Zhengzhou, 450000 Henan China; 2 Moorfields Eye Hospital NHS Foundation Trust, London, UK; 3 UCL Institute of Ophthalmology, University College London, London, UK; 4 State Key Laboratory of Ophthalmology, Optometry and Vision Science, Wenzhou Medical University, Wenzhou, China

**Keywords:** Diseases, Health care, Risk factors

## Abstract

To compare early diabetic retinopathy (DR) severity level and the abilities in detecting early DR lesions among conventional five-field, ultrawide-field (UWF) Optos, and UWF Clarus fundus imaging methods. This was a single-center, prospective, clinic-based, and comparative study. In total, 157 consecutive patients with diabetes mellitus were enrolled in this study. All patients underwent comprehensive ophthalmological examinations. Following mydriasis, each eye was examined with conventional five-field, UWF Optos, and UWF Clarus fundus imaging methods. The initial UWF images were overlaid with a template mask that obscured the retina, which created a five-field view from UWF images (covered UWF images). The covered UWF images were then graded, after which the template mask was removed, and the original UWF images were also evaluated. All images were graded using the International Clinical DR severity scale. DR grades were compared and analyzed by weighted kappa statistics among the three fundus imaging methods. In total, 157 consecutive patients with diabetes (302 eyes) were enrolled in this study. Weighted kappa statistics for agreement were 0.471 (five-field vs. covered Optos), 0.809 (five-field vs. covered Clarus), 0.396 (covered Optos vs. covered Clarus), 0.463 (five-field vs. Optos), 0.521 (five-field vs. Clarus 133°), 0.500 (five-field vs. Clarus 200°), 0.323 (Optos vs. Clarus 133°), and 0.349 (Optos vs. Clarus 200°). The area under curve of covered Clarus images was higher than that of conventional five-field images at three different thresholds. Compared with conventional five-field and Optos fundus imaging methods, Clarus fundus imaging methods exhibited excellent performance in assessing early DR severity. Thus, Clarus fundus imaging methods were superior for early detection of DR.

## Introduction

Diabetic retinopathy (DR) is a primary cause of preventable vision loss among working-aged adults in developing countries^1–3^. However, before patients with diabetes reach an advanced stage of DR, they usually remain asymptomatic. Therefore, to avoid visual loss, regular screening and early detection of vision-threatening DR in patients with diabetes are necessary to ensure timely treatment^4–6^.

At present, the Early Treatment Diabetic Retinopathy Study (ETDRS) fundus photography protocol remains the “gold standard” for determining DR severity; it requires pharmacological mydriasis to acquire seven individual fields in a stereoscopic manner, thereby generating 14 retinal images^7^. The ETDRS seven-field imaging can cover up to approximately 75° of the retina, which represents approximately 30% of the entire retinal area^8,9^. However, it requires additional time to obtain, trained personnel for both imaging and grading, and good cooperation between patients and photographers. The ETDRS severity scale is also regarded as the “gold standard” for grading the severity of DR in clinical trials. However, the photographic grading system has more levels than are required for clinical care, and the specific definitions of the levels are detailed and require comparison with standard photographs. Thus, it is difficult to remember and apply in clinical practice. In previous studies, fundus imaging covering up to 45°–60° of the retina had substantial and almost perfect agreement with standard ETDRS seven-field imaging covering up to 75° of the retina^10–12^. Therefore, to improve fundus photography and DR grading efficiency, five-field imaging covering approximately 60° of the retina, which represents approximately 20% of the entire retinal area, has been investigated^13^. Considering the increasing number of patients with diabetes and the awareness of diabetes eye care, there is a need for methods other than the time-consuming conventional screening approach, which covers a small retinal area. Advances in retinal imaging technology now allow ultrawide-field (UWF) fundus imaging to evaluate larger areas of the retinal surface in a single image^14^. The UWF Optos fundus imaging device was designed to capture up to 200° in a single image without mydriasis and achieve a resolution of 14 µm. However, this device has several limitations. In particular, it demonstrates inherent distortion and color variation, which cause occasional slight differences from real color images. Small retinal hemorrhages and microaneurysms are reportedly more difficult to identify in Asian individuals, it remains unclear whether Optos imaging is suitable for early DR screening^15^. The new UWF Clarus fundus imaging device can capture up to 133° of retina in a single image without mydriasis and 200° of retina in four mydriatic montage images (superior, inferior, nasal, and temporal periphery). This fundus imaging device uses a combination of red, green, and blue images to provide a realistic color fundus image. The high resolution and high imaging quality allow detection of various lesions in the retina.

At present, one study has compared DR severity assessment between Optos and Clarus fundus imaging methods. In the study, 40 of 46 eyes were proliferative DR (PDR) according to ETDRS severity level^16^. To our knowledge, no study has compared the validities of these three retinal imaging methods for assessing early DR severity. In this study, we investigated the validities of these retinal imaging methods for assessing early DR severity.

## Methods

### Participants

This single-center, prospective, clinic-based, comparative study adhered to the tenets of the Declaration of Helsinki and was approved by the ethics committee of Henan Eye Hospital. Before ophthalmic examinations, all patients provided written informed consent. This study enrolled consecutive patients with diabetes mellitus who visited Henan Eye Hospital from September 2020 to April 2021.

Patient eligibility was determined from review of medical records for patients who were diagnosed with diabetes mellitus in accordance with World Health Organization criteria; this study was designed to evaluate early DR severity level. To evaluate the abilities in detecting early DR lesions, recruitment was weighted toward less severe levels of DR. Therefore, DR severity levels ranged from no DR to severe NPDR based on five-field fundus image grading according to the International Clinical DR severity scale. The inclusion criteria were as follows: age 19–75 years, diagnosis of types 1 or 2 diabetes mellitus, ≥ 1 year since diagnosis with diabetes mellitus, and consent to undergo imaging. Exclusion criteria were as follows: inability to cooperate during retinal imaging or clinical examination; diagnosis of PDR; a history of contraindications to mydriasis (e.g., angle-closure glaucoma); a history of severe media opacity that may obscure the retina (e.g., severe cataract); a history of clinically significant fundus abnormalities other than DR (e.g., central retina vein occlusion); a history of eye surgery or panretinal photocoagulation; and/or ongoing pregnancy. All patients underwent comprehensive ophthalmic examinations by retinal specialists, including standardized logMAR visual acuity assessment, intraocular pressure, and slit-lamp biomicroscopy.

### Imaging acquisition

For baseline images, all patients underwent pupillary dilation. They then underwent a single session of fundus imaging for each eye with conventional five-field and UWF fundus imaging methods; images were collected by an experienced imager. The five-field view (one 30° image of the macula and four 30° images of the peripheral retina combined into a mosaic image) was collected with the Zeiss FF4 fundus imaging device (Carl Zeiss, Jena, Germany)^13^. The UWF fundus imaging method included a single 200° image using the Optos (Daytona P200T, Optos PLC, Dunfermline, United Kingdom), as well as a single 133° image and a 200° image consisting of four mydriatic montage images using the Clarus (Clarus 500, Carl Zeiss Meditec AG, Jena, Germany).

### Imaging grading

**Figure 1:**
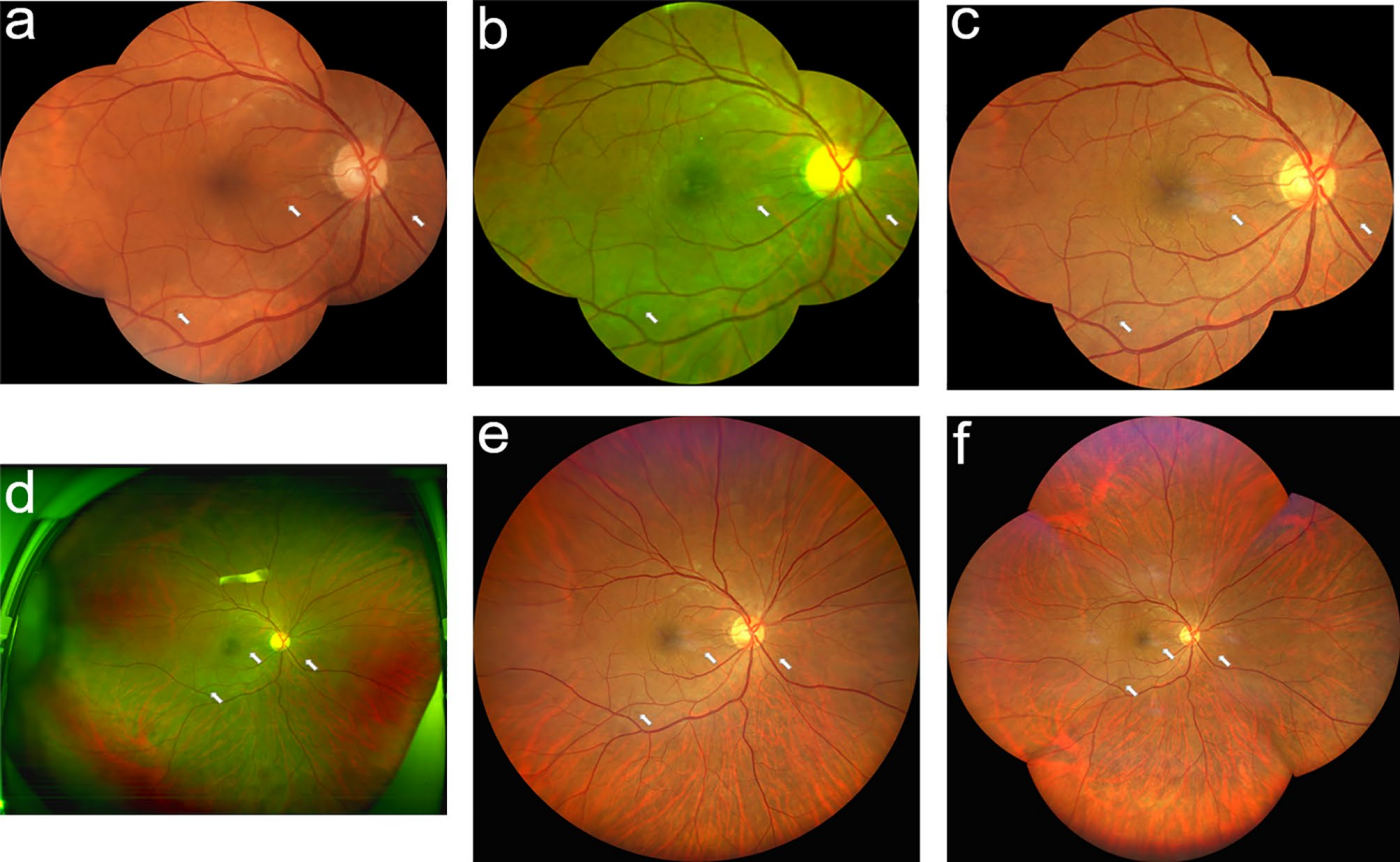
Three fundus imaging systems: (**a**) Conventional five-field fundus imaging; (**b**) Covered Optos fundus imaging; (**c**) Covered Clarus fundus imaging; (**d**) Optos fundus imaging; (**e**) Clarus 133° fundus imaging; (**f**) Clarus 200° fundus imaging. Arrow indicates microaneurysms or hemorrhage. Notably, the microaneurysms or hemorrhage is difficult to identify because of lower contrast for Optos fundus imaging.

For evaluation of DR severity, all images were uploaded to a display that could adjust brightness, contrast, and gamma using a standardized procedure^17^. All images were independently graded by two trained senior retinal doctors who were masked to patient data; grading was performed in accordance with the proposed International Clinical Diabetic Retinopathy severity scale^18^. To determine the reproducibility of grading for each system in the detection of DR lesions, we also evaluated the weighted kappa of DR severity between specialists for three fundus imaging methods. For all patients, each eye was separately graded using six images (five-field view, covered Optos, covered Clarus, Optos, Clarus 133°, and Clarus 200°). Any grading disagreements between the two senior readers were resolved by a trained senior ophthalmologist; therefore, a single outcome was recorded for each image. While UWF images were graded, they were initially presented with a template mask, which obscured the retina with an automatic digital overlay that only displayed the five-field view from UWF images (five-field image, Fig. 1a; covered Optos image, Fig. 1b; and covered Clarus image, Fig. 1c).When DR grading using covered images was completed, the template mask was removed and UWF-uncovered images were evaluated (Optos image, Fig. 1d; Clarus 133° image, Fig. 1e; and Clarus 200° image, Fig. 1f). To evaluate the abilities of two real-color fundus imaging methods (five-field vs. Clarus) to identify subtle lesions of DR, a senior retinal specialist independently conducted direct side-by-side comparisons for all images in which the DR severity differed between five-field and covered Clarus images. A single final result was then generated for each eye; this was regarded as the grading standard.

### Statistical analyses

Pairwise grading agreements between methods and between specialists were determined by calculating weighted kappa coefficients. Statistical analyses were performed using the Landis and Koch guidelines for interpretation of kappa statistics (0.0–0.2, slight agreement; 0.21–0.40, fair agreement; 0.41–0.60, moderate agreement; 0.61–0.80, substantial agreement; and 0.81–1.00, almost perfect agreement)^19^. Sensitivity, specificity, positive predictive value, negative predictive value and the area under curve (AUC) were determined using the receiver operating characteristic curve. All analyses were conducted using SPSS statistical software (version 26.0).

### Ethics declarations

This study adhered to the Tenets of the Declaration of Helsinki and was approved by the ethics committee of Henan Eye Hospital (HNEECKY-202005). Each participant provided informed consent after receiving a detailed explanation of the procedure before treatment. In this study, patient-identifiable data were hidden.

## Results

### Characteristics

In this study, 169 patients (314 eyes) completed examinations with the three fundus imaging methods; 12 eyes were excluded because of PDR (n = 4), central retinal vein occlusion (n = 2), panretinal photocoagulation (n = 4), and ungradable Optos images (n = 2). In total, 157 patients with diabetes (302 eyes) were enrolled. Patient characteristics and early DR severity distributions are summarized in Table 1.

**Table 1:** Study participant characteristics and DR severity distributions.

Baseline patient demographics (N = 157 patients)	Mean ± standard deviation (range) or N (%)
Age (years)	54.4 ± 9.9 (21–75)
Female/male	50 (31.8%)/107 (68.2%)
Duration of DR (years)	9.67 ± 6.55 (1–28)
HbA_1c_ (%)	8.56 ± 5.37 (5.6–13.3)
Body mass index (kg/m^2^)	24.7 ± 2.48 (19.2–32.7)
Intraocular pressure (mmHg)	16.47 ± 1.98 (12.1–22.5)
Baseline logMAR visual acuity	0.08 ± 0.20 (0–1.4)
Retinopathy severity	
No DR	174 (57%)
Mild NPDR	75 (25%)
Moderate NPDR	45 (15%)
Severe NPDR	8 (3%)

*DR* diabetic retinopathy, *logMAR* logarithm of the minimum angle of resolution, *NPDR* non-proliferative diabetic retinopathy.

### Comparison of the reproducibility of grading for each system during DR severity assessment

Concerning DR severity, the level of agreement between the two specialists was almost perfect with weighted kappa of 0.921 (95% confidence interval [CI], 0.885–0.958) and 14 eyes requiring arbitration for five-field images, almost perfect with weighted kappa of 0.835 (95% CI, 0.769–0.898) and 20 eyes requiring arbitration for covered Optos images, almost perfect with weighted kappa of 0.915 (95% CI, 0.867–0.954) and 16 eyes requiring arbitration for covered Clarus images, almost perfect with weighted kappa of 0.818 (95% CI, 0.755–0.880) and 27 eyes requiring arbitration for Optos images, almost perfect with weighted kappa of 0.931 (95% CI, 0.888–0.966) and 14 eyes requiring arbitration for Clarus 133° images, and almost perfect with weighted kappa of 0.918 (95% CI, 0.879–0.952) and 17 eyes requiring arbitration for Clarus 200° images.

### Comparison of DR severity among images with same fields (conventional five-field, covered Optos, and covered Clarus fundus imaging methods)

**Figure 2:**
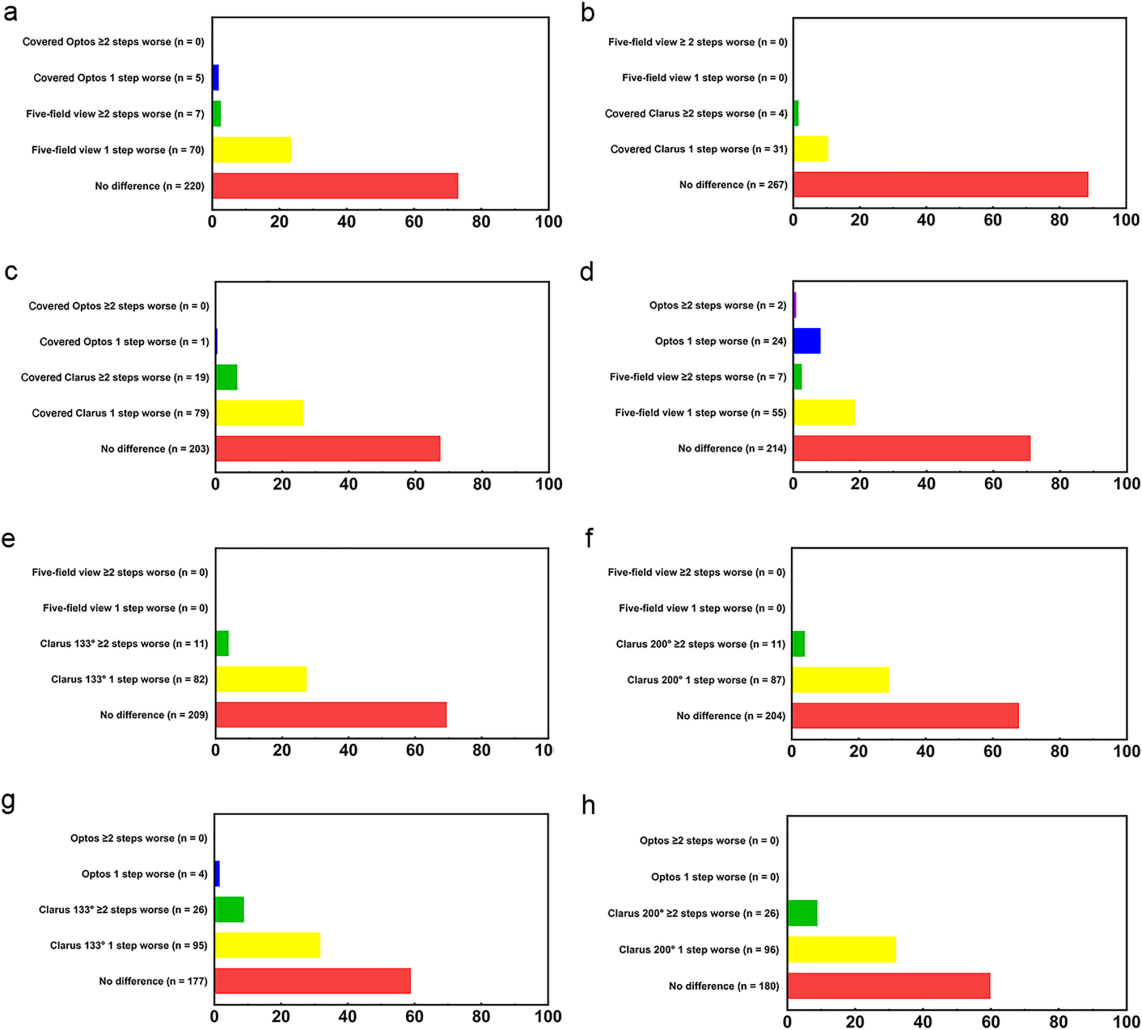
Differences in DR severity based on Proposed International Clinical Diabetic Retinopathy severity scale. (**a**) No difference accounted for 220 eyes (72.8%) of the total; Covered Optos imaging 1 step worse, 5 eyes (1.7%); Five-field imaging 1 step worse, 70 eyes (23.2%); Five-field imaging 2 or more steps worse, 2.3% (7 eyes). (**b**) No difference accounted for 267 eyes (88.4%) of the total; Covered Clarus imaging 1 step worse, 31 eyes (10.3%); Covered Clarus imaging 2 or more steps worse, 4 eyes (1.3%). (**c**) No difference accounted for 203 eyes (67.2%) of the total; Covered Clarus imaging 1 step worse, 79 eyes (26.2%); Covered Clarus imaging 2 or more steps worse, 19 eyes (6.3%); Covered Optos imaging 1 step worse, 1 eye (0.3%). (**d**) No difference accounted for 214 eyes (70.9%) of the total; Optos imaging 1 step worse, 24 eyes (8.0%); Optos imaging 2 or more steps worse, 2 eyes (0.6%); Five-field imaging 1 step worse, 55 eyes (18.2%); Five-field imaging 2 or more steps worse, 7 eyes (2.3%). (**e**) No difference accounted for 209 eyes (69.2%) of the total; Clarus 133° imaging 1 step worse, 82 eyes (27.2%); Clarus 133° imaging 2 or more steps worse, 11 eyes (3.6%). (**f**) No difference accounted for 204 eyes (67.6%) of the total; Clarus 200° imaging 1 step worse, 87 eyes (28.8%); Clarus 200° imaging 2 or more steps worse, 11 eyes (3.6%). (**g**) No difference accounted for 177 eyes (58.6%) of the total; Clarus 133° imaging 1 step worse, 95 eyes (31.5%); Clarus 133° imaging 2 or more steps worse, 26 eyes (8.6%); Optos imaging 1 step worse, 4 eyes (1.3%). (**h**) No difference accounted for 180 eyes (59.6%) of the total; Clarus 200° imaging 1 step worse, 96 eyes (31.8%); Clarus 133° imaging 2 or more steps worse, 26 eyes (8.6%).

Comparison of DR severity between five-field and covered Optos imaging methods showed exact matches in 220 eyes (72.8%) with moderate agreement (weighted kappa agreement, 0.471 [95% CI, 0.376–0.558]; eTable 1, Fig. 2a). Seventy-seven eyes (25.5%) exhibited one or more DR levels worse on five-field images. Only five eyes (1.7%) had DR one level worse on covered Optos images. Comparison of DR severity between five-field and covered Clarus imaging methods showed exact matches in 267 eyes (88.4%) with almost perfect agreement (weighted kappa agreement, 0.809 [95% CI, 0.750–0.864]; eTable 2, Fig. 2b). Thirty-five eyes (11.6%) exhibited one or more DR levels worse on covered Clarus images. Comparison of DR severity between covered Optos and covered Clarus imaging methods showed exact matches in 203 eyes (67.2%) with fair agreement (weighted kappa agreement, 0.396 [95% CI, 0.318–0.480], eTable 3, Fig. 2c). Ninety-eight eyes (32.5%) exhibited one or more DR levels worse on covered Clarus images. Only one eye (0.3%) had DR one level worse on covered Optos images.

### Comparison of DR severity among conventional five-field, Optos, and Clarus fundus imaging methods

Comparison of DR severity between five-field and Optos imaging methods showed exact matches in 214 eyes (70.9%) with moderate agreement (weighted kappa agreement, 0.463 [95% CI, 0.383–0.544]; eTable 4, Fig. 2d). Sixty-two eyes (20.5%) exhibited one or more DR levels worse on five-field images; 26 eyes (8.6%) exhibited one or more DR levels worse on Optos images. Comparison of DR severity between five-field and Clarus 133° imaging methods showed exact matches in 209 eyes (69.2%) with moderate agreement (weighted kappa agreement, 0.521 [95% CI, 0.455–0.585]; eTable 5, Fig. 2e). Ninety-three eyes (30.8%) exhibited one or more DR levels worse on Clarus 133° images. Comparison of DR severity between five-field and Clarus 200° imaging methods showed exact matches in 204 eyes (67.5%) with moderate agreement (weighted kappa agreement, 0.500 [95% CI, 0.431–0.575]; eTable 6, Fig. 2f). Ninety-eight eyes (32.5%) exhibited one or more DR levels worse on Clarus 200° images. Comparison of DR severity between Optos and Clarus 133° imaging methods showed exact matches in 177 eyes (58.6%) with fair agreement (weighted kappa agreement, 0.323 [95% CI, 0.245–0.401]; eTable 7, Fig. 2g). One hundred twenty-one eyes (40.1%) exhibited one or more DR levels worse on Clarus 133° images; only four eyes (1.3%) had DR one level worse on Optos images. Comparison of DR severity between Optos and Clarus 200° imaging methods showed exact matches in 180 eyes (59.6%) with fair agreement (weighted kappa agreement, 0.349 [95% CI, 0.276–0.423]; eTable 8, Fig. 2h). One hundred twenty-two eyes (40.4%) exhibited one or more DR levels worse on Clarus 200° images.

### Comparison of accuracy in identifying subtle DR lesions between five-field and Clarus fundus imaging methods

The DR severity level differed between five-field and covered Clarus images for 35 eyes (11.6%). The prevalences of DR severity level determined by the grading standard, five-field, and covered Clarus images for each eye are summarized in eTable 9. Sensitivity, specificity, positive predictive value, negative predictive value, and AUC of five-field and covered Clarus images are shown in eTables 10 and 11. The AUC of covered Clarus images was higher than the AUC of five-field images at three different thresholds.

## Discussion

In this study, the level of agreement between the two graders concerning DR severity was almost perfect for all three fundus imaging systems, which indicated good reproducibility of grading for each system. Our study found moderate agreement between five-field and covered Optos fundus images, such that covered Optos images tended to show a lower DR severity level. As reported previously, the DRCR Retina Network Protocol AA report and several single-center studies have suggested moderate to substantial agreement between the standard ETDRS seven fields and Optos fundus images^5,20,21^. Potential reasons for this difference are as follows. First, the combination of monochromatic red and green scanning laser ophthalmoscopy methods results in some differences between Optos images and real color images^22^. Second, it exhibits a resolution of 14 µm, which is lower than the resolution of five-field images. Third, Asian eyes exhibit narrower interpalpebral fissures and darker pigmented fundi that may increase the likelihood of inferior image quality; these aspects may prevent clear images of retinal lesions^15^.

Our results indicated that five-field images had almost perfect agreement with covered Clarus images. In total, 35 eyes (11.6%) for the DR severity level did not match, six eyes (2.0%) for five-field images and 29 eyes (9.6%) for covered Clarus images were considered more accurate after side-by-side image comparisons. The AUC of covered Clarus images was higher than the AUC of five-field images at three different thresholds. The reason could be that Clarus images were formed by combining red, green, and blue images, thereby generating realistic color images^16,23^. Additionally, red scanning laser ophthalmoscopy penetrates deep into the choroid, such that Clarus images were not easily affected by cataracts^23^. Furthermore, Clarus images had 7-µm resolution that allowed the detection of microaneurysms and small retinal hemorrhages. Therefore, Clarus fundus imaging methods can detect more subtle DR lesions. The factors mentioned above may also explain the fair agreement between covered Clarus and covered Optos images.

Comparison of five-field and Optos images revealed discrepancies of one or more severity levels in 26 eyes (8.6%) in Optos images. However, 62 eyes (20.5%) in five-field images showed discrepancies of one or more DR severity levels. These discrepancies may have been caused by the above-mentioned inability to detect subtle lesions in Optos images. Comparison of five-field images with Clarus images showed moderate agreement; discrepancies of one or more DR severity levels were observed in Clarus images. A greater proportion of eyes had a higher DR severity level on Clarus and Optos images than on five-field images, especially on Clarus images. These data suggest that UWF fundus imaging technology can detect more peripheral lesions, which leads to an increased DR severity level. Some previous studies have shown that eyes with peripheral lesions have a more than threefold increased risk of DR progression ≥ 2 steps and an almost fivefold increased risk of progression to proliferative DR^9,22^. Although the roles of these additional lesions in risk determination and clinical significance have not been identified because it lacked a large number of patients with PDR, these data suggest that the peripheral lesions on UWF images might be useful for identifying more severe DR and providing clearer clinical assessment.

DR severity showed fair agreement between Optos and Clarus 133°/200° images. Although Optos images captured greater retinal areas than did Clarus 133° images and the same retinal area as did Clarus 200° images, discrepancies of one or more DR severity levels were mostly observed in Clarus images. In addition to the reasons mentioned above, the Clarus imaging system has a partially confocal optics feature that may limit the presence of eyelash and eyelid artifacts in retinal images^16^. Thus, the Clarus imaging system captured a larger visible retinal area.

The notable strength of this study was that it was the first prospective study to evaluate early DR severity using the conventional five-field, Optos, and Clarus images in a large cohort of patients with diabetes. However, there were a few limitations. First, this study was performed in a single institution, rather than multiple institutions. Second, this study used the five-field images as the standard for comparison, rather than the gold standard ETDRS method. Third, there might have been bias related to the graders because the camera could not be masked. Besides, this study evaluated early DR severity using the International Clinical Diabetic Retinopathy severity scale that is widely used to assess DR severity in clinical practice, rather than the ETDRS severity scale that was designed primarily for clinical trials. Thus, the results are more applicable to assessment of DR in clinical practice, rather than in clinical trials. These limitations will be addressed in our future studies.

In conclusion, compared with five-field and Optos, the Clarus fundus imaging methods, with its high resolution and high-quality wide field fundus, exhibited excellent performance in assessing early DR severity. Thus, Clarus fundus imaging methods was superior for early detection of DR.

### Supplementary Information

Supplementary Tables.

## Data Availability

The datasets used and/or analyesd during the current study are available from the corresponding author on reasonable request.

## Abbreviations

DR: Diabetic retinopathy
ETDRS: Early Treatment Diabetic Retinopathy Study
UWF: Ultrawide-field
PDR: Proliferative diabetic retinopathy
NPDR: Non-proliferative diabetic retinopathy
AUC: The area under curve
CI: Confidence interval
logMAR: Logarithm of the minimum angle of resolution
