# Supplementary material for: Assessment of early diabetic retinopathy severity using ultra-widefield Clarus versus conventional five-field and ultra-widefield Optos fundus imaging
Source: Sci Rep. 2023 Oct 10;13:17131. doi: 10.1038/s41598-023-43947-5 (PMC10564714; doi:10.1038/s41598-023-43947-5)
Supplement: Supplementary file 1 — Supplementary Tables. [file 41598_2023_43947_MOESM1_ESM.docx]

**Supplementary Online Materials**

**eTable 1. DR Grading Comparing Five-field Imaging with** **Covered Optos Imaging**

**eTable 2. DR Grading Comparing Five-field Imaging with Covered Clarus Imaging**

**eTable 3. DR Grading Comparing** **Covered** **Optos Imaging with Covered Clarus Imaging**

**eTable 4. DR Grading Comparing Five-field Imaging with** **Optos Imaging**

**eTable 5. DR Grading Comparing Five-field Imaging with Clarus 133° Imaging**

**eTable 6. DR Grading Comparing Five-field Imaging with** **Clarus 200° Imaging**

**eTable 7. DR Grading Comparing** **Optos Imaging with Clarus 133° Imaging**

**eTable 8. DR Grading Comparing Optos Imaging with Clarus 200° Imaging**

**eTable 9. DR Grading of Five‑Field, Covered Clarus Imaging and Grading Standard**

**eTable 10. Accuracy of Five-Field Images for Detecting Any Diabetic Retinopathy**

**eTable 11. Accuracy of Covered Clarus for Detecting Any Diabetic Retinopathy**

**eTable 1. DR Grading Comparing Five-field Imaging with Covered Optos Imaging**

|  | **Covered Optos imaging** | | | | | |
| --- | --- | --- | --- | --- | --- | --- |
| **Five-field imaging** | **No DR** | **Mild**  **NPDR** | **Moderate**  **NPDR** | **Severe**  **NPDR** | **Total** | **Weighted Kappa (95% CI)** |
| No DR | 172 | 2 | 0 | 0 | 174 | 0.471  (0.376-0.558) |
| Mild NPDR | 53 | 19 | 3 | 0 | 75 |  |
| Moderate NPDR | 7 | 14 | 24 | 0 | 45 |  |
| Severe NPDR | 0 | 0 | 3 | 5 | 8 |  |
| Total | 232 | 35 | 30 | 5 | 302 |  |

Abbreviations: DR, diabetic retinopathy; NPDR, non-proliferative diabetic retinopathy; CI, confidence interval.

**eTable 2. DR Grading Comparing Five-field Imaging with Covered Clarus Imaging**

|  | **Covered Clarus imaging** | | | | | | |
| --- | --- | --- | --- | --- | --- | --- | --- |
| **Five-field imaging** | **No DR** | **Mild**  **NPDR** | **Moderate**  **NPDR** | **Severe**  **NPDR** | **Total** | **Weighted Kappa**  **(95% CI)** | |
| No DR | 162 | 10 | 2 | 0 | 174 | | 0.809  (0.750-0.864) |
| Mild NPDR | 0 | 61 | 12 | 2 | 75 | |  |
| Moderate NPDR | 0 | 0 | 36 | 9 | 45 | |  |
| Severe NPDR | 0 | 0 | 0 | 8 | 8 | |  |
| Total | 162 | 71 | 50 | 19 | 302 | |  |

Abbreviations: DR, diabetic retinopathy; NPDR, non-proliferative diabetic retinopathy; CI, confidence interval.

**eTable 3. DR Grading Comparing** **Covered Optos Imaging with Covered Clarus Imaging**

|  | **Covered Clarus imaging** | | | | | |
| --- | --- | --- | --- | --- | --- | --- |
| **Covered Optos imaging** | **No DR** | **Mild**  **NPDR** | **Moderate**  **NPDR** | **Severe**  **NPDR** | **Total** | **Weighted Kappa (95% CI)** |
| No DR | 161 | 56 | 13 | 2 | 232 | 0.396  (0.318-0.480) |
| Mild NPDR | 1 | 15 | 15 | 4 | 35 |  |
| Moderate NPDR | 0 | 0 | 22 | 8 | 30 |  |
| Severe NPDR | 0 | 0 | 0 | 5 | 5 |  |
| Total | 162 | 71 | 50 | 19 | 302 |  |

Abbreviations: DR, diabetic retinopathy; NPDR, non-proliferative diabetic retinopathy; CI, confidence interval.

**eTable 4. DR Grading Comparing Five-field Imaging with Optos Imaging**

|  | **Optos imaging** | | | | | |
| --- | --- | --- | --- | --- | --- | --- |
| **Five-field imaging** | **No DR** | **Mild**  **NPDR** | **Moderate**  **NPDR** | **Severe**  **NPDR** | **Total** | **Weighted Kappa (95% CI)** |
| No DR | 159 | 13 | 2 | 0 | 174 | 0.463  (0.383-0.544) |
| Mild NPDR | 44 | 22 | 9 | 0 | 75 |  |
| Moderate NPDR | 7 | 9 | 27 | 2 | 45 |  |
| Severe NPDR | 0 | 0 | 2 | 6 | 8 |  |
| Total | 210 | 44 | 40 | 8 | 302 |  |

Abbreviations: DR, diabetic retinopathy; NPDR, non-proliferative diabetic retinopathy; CI, confidence interval.

**eTable 5. DR Grading Comparing Five-field Imaging with Clarus 133° Imaging**

|  | **Clarus 133° imaging** | | | | | |
| --- | --- | --- | --- | --- | --- | --- |
| **Five-field imaging** | **No DR** | **Mild**  **NPDR** | **Moderate**  **NPDR** | **Severe**  **NPDR** | **Total** | **Weighted Kappa (95% CI)** |
| No DR | 144 | 24 | 6 | 0 | 174 | 0.521  (0.455-0.585) |
| Mild NPDR | 0 | 43 | 27 | 5 | 75 |  |
| Moderate NPDR | 0 | 0 | 14 | 31 | 45 |  |
| Severe NPDR | 0 | 0 | 0 | 8 | 8 |  |
| Total | 144 | 67 | 47 | 44 | 302 |  |

Abbreviations: DR, diabetic retinopathy; NPDR, non-proliferative diabetic retinopathy; CI, confidence interval.

**eTable 6. DR Grading Comparing Five-field Imaging with Clarus 200° Imaging**

|  | **Clarus 200° imaging** | | | | | |
| --- | --- | --- | --- | --- | --- | --- |
| **Five-field imaging** | **No DR** | **Mild**  **NPDR** | **Moderate**  **NPDR** | **Severe**  **NPDR** | **Total** | **Weighted Kappa (95% CI)** |
| No DR | 139 | 29 | 6 | 0 | 174 | 0.500  (0.431-0.575) |
| Mild NPDR | 0 | 43 | 27 | 5 | 75 |  |
| Moderate NPDR | 0 | 0 | 14 | 31 | 45 |  |
| Severe NPDR | 0 | 0 | 0 | 8 | 8 |  |
| Total | 139 | 72 | 47 | 44 | 302 |  |

Abbreviations: DR, diabetic retinopathy; NPDR, non-proliferative diabetic retinopathy; CI, confidence interval.

**eTable 7. DR Grading Comparing Optos Imaging with Clarus 133° Imaging**

|  | **Clarus 133° imaging** | | | | | |
| --- | --- | --- | --- | --- | --- | --- |
| **Optos imaging** | **No DR** | **Mild NPDR** | **Moderate NPDR** | **Severe NPDR** | **Total** | **Weighted Kappa (95% CI)** |
| No DR | 140 | 50 | 18 | 2 | 210 | 0.323  (0.245-0.401) |
| Mild NPDR | 4 | 17 | 17 | 6 | 44 |  |
| Moderate NPDR | 0 | 0 | 12 | 28 | 40 |  |
| Severe NPDR | 0 | 0 | 0 | 8 | 8 |  |
| Total | 144 | 67 | 47 | 44 | 302 |  |

Abbreviations: DR, diabetic retinopathy; NPDR, non-proliferative diabetic retinopathy; CI, confidence interval.

**eTable 8. DR Grading Comparing Optos Imaging with Clarus 200° Imaging**

|  | **Clarus 200° imaging** | | | | | |
| --- | --- | --- | --- | --- | --- | --- |
| **Optos imaging** | **No DR** | **Mild**  **NPDR** | **Moderate**  **NPDR** | **Severe**  **NPDR** | **Total** | **Weighted Kappa (95% CI)** |
| No DR | 139 | 51 | 18 | 2 | 210 | 0.349  (0.276-0.423) |
| Mild NPDR | 0 | 21 | 17 | 6 | 44 |  |
| Moderate NPDR | 0 | 0 | 12 | 28 | 40 |  |
| Severe NPDR | 0 | 0 | 0 | 8 | 8 |  |
| Total | 139 | 72 | 47 | 44 | 302 |  |

Abbreviations: DR, diabetic retinopathy; NPDR, non-proliferative diabetic retinopathy; CI, confidence interval.

**eTable 9. DR Grading of Grading Standard, Five‑Field** **and Covered Clarus Imaging**

| **DR severity level** | **Grading standard** | **Five-field imaging** | **Covered Clarus imaging** |
| --- | --- | --- | --- |
| No DR | 166 (55.0%) | 174 (57.6%) | 162 (53.6%) |
| Mild NPDR | 68 (22.5%) | 75 (24.8%) | 71 (23.5%) |
| Moderate NPDR | 50 (16.5%) | 45 (14.9%) | 50 (16.6%) |
| Severe NPDR | 18 (6.0%) | 8 (2.7%) | 19 (6.3%) |

Abbreviations: DR, diabetic retinopathy; NPDR, non-proliferative diabetic retinopathy;

**eTable 10. Accuracy of Five-Field Imaging for Detecting Any Diabetic Retinopathy**

| **Threshold** | **Sensitivity**  **(95% CI)** | **Specificity**  **(95% CI)** | **Positive Predictive Value** | **Negative Predictive Value** | **AUC**  **(95% CI)** |
| --- | --- | --- | --- | --- | --- |
| DR absent VS.  DR Present | 1.000  (0.998-1.000) | 0.976  (0.952-0.999) | 0.971 | 1.000 | 0.971  (0.947-0.974) |
| Mild NPDR or better VS. Moderate NPDR or worse | 0.779  (0.678-0.881) | 1.000  (0.997-1.000) | 1.000 | 0.940 | 0.956  (0.925-0.987) |
| Moderate NPDR or better VS. Severe NPDR | 0.444  (0.190-0.699) | 1.000  (0.990-1.000) | 1.000 | 0.996 | 0.942  (0.900-0.984) |

Abbreviations: DR, diabetic retinopathy; NPDR, non-proliferative diabetic retinopathy; CI, confidence interval. AUC, the area under curve;

**eTable 11. Accuracy of Covered Clarus Imaging for Detecting Any Diabetic Retinopathy**

| **Threshold** | **Sensitivity**  **(95% CI)** | **Specificity**  **(95% CI)** | **Positive Predictive Value** | **Negative Predictive Value** | **AUC**  **(95% CI)** |
| --- | --- | --- | --- | --- | --- |
| DR absent VS.  DR Present | 0.941  (0.901-0.981) | 1.000  (0.998-1.000) | 1.000 | 0.954 | 0.994  (0.987-1.000) |
| Mild NPDR or better VS. Moderate NPDR or worse | 1.000  (0.997-1.000) | 0.996  (0.987-1.000) | 0.986 | 1.000 | 0.998  (0.995-1.000) |
| Moderate NPDR or better VS. Severe NPDR | 1.000  (0.996-1.000) | 0.996  (0.990-1.000) | 0.947 | 1.000 | 0.998  (0.994-1.000) |

Abbreviations: DR, diabetic retinopathy; NPDR, non-proliferative diabetic retinopathy; CI, confidence interval. AUC, the area under curve;
